# Identification and in silico analysis of noval alteration Arg420Gly in KIT proto oncogene among acute myeloid leukemia patients

**DOI:** 10.1038/s41598-022-23934-y

**Published:** 2022-11-10

**Authors:** Afia Muhammad Akram, Mubashir Hassan, Asma Chaudhary, Sikandar Hayat, Qurban Ali, Taha Hussain, Amjad Zafar, Muhammad Arshad Javed

**Affiliations:** 1 Department of Zoology, Division of Science and Technology, University of Education, Township Lahore, Pakistan; 2 Institute of Molecular Biology and Biotechnology, The University of Lahore, Lahore, Pakistan; 3 Department of Plant Breeding and Genetics, Faculty of Agricultural Sciences, University of the Punjab, Lahore, Pakistan; 4 Department of Oncology, Mayo Hospital, Anarkali Bazar, Lahore, Pakistan

**Keywords:** Cancer, Oncogenes

## Abstract

A number of studies have reported frequent incidence of c-kit gene mutations in association with core binding factor acute myeloid leukemia (CBF-AML). These genetic changes have become important prognostic predictors in patients with abnormal karyotype. Aim of this study was the detection of nucleotide alterations in newly diagnosed acute myeloid leukemia patients for three exons of c-kit gene, including cytogenetically normal patients. Thirty-one de novo AML patients were screened for any possible variations in exon 8, 11 and 17 sequences of c-kit proto-oncogene leading to amino acid substitutions or frame shift. Sanger sequencing method was employed followed by sequence analysis. Mutation data was then correlated with clinical and hematological parameters of patients and prognostic significance of genetic changes was assessed as well. The computational tools were then used to further understand the extent of damage caused by these mutations to c-kit protein. Fifteen (48.4%) mutant patients were observed with single, double or multiple mutations in one, two or all three exons studied. The analysis revealed eight new alterations which were not reported previously. Significant variation among mutant and non-mutant group of patients was observed with respect to FAB subtypes (x^2^ = 12.524, *p* = 0.029), Spleen size (x^2^ = 4.288, *p* = 0.038) and Red blood cell count (x^2^ = 8.447, *p* = 0.007). The survival analysis indicates poor overall and event free survival outcomes in mutant individuals. Furthermore, the in silico analysis suggests that changes in nucleotide sequences can possibly damage the protein structure and effect it’s function. This study emphasizes the need to consider screening of c-kit gene alterations not only in CBF-AML but in cytogenetically normal AML patients as well. In current investigation the effect of mutation Arg420Gly on structure and function of c-kit protein was investigated, as this was the most observed substitution in present cohort. Various bioinformatics tools and techniques were employed, which determined that Arg420Gly is possibly non-pathogenic mutation.

## Introduction

Human c-kit, the cellular counterpart of v-kit which is derived from sarcoma virus Hardy-Zuckerman 4-feline, encodes a 145KD receptor tyrosine kinase (RTK) and is positioned on chromosome 4q11-q12. Activation of the c-kit gene is made possible by ligands and stem cell factors (SCF), playing important roles in melanogenesis, hematopoiesis and spermatogenesis^1,2^. C-kit gene is commonly known as proto-oncogene and activating c-kit mutations may possibly be involved in establishing leukemia^3^. More importantly, a number of reports state occurrence of mutations in Core binding factor acute myeloid leukemia (CBF-AML)^4,5^. This gene codes for a transmembrane receptor kinase which normally expresses in germ cells, mast cells, neural crest-derived melanocytes and hematopoietic stem cells. C-kit gene in humans is mapped at chromosome 4q12 adjoining the platelet derived growth factor receptor (PDGFR), which is highly homologous^6^. Binding of KIT ligand to its receptor is significant for activation of downstream signaling passageways which is vital for cell propagation, distinction, and existence^5,7,8^. When mutation occurs in c-KIT gene, it activates without binding its ligand and cause abnormal hematopoietic stem cells formation^9^. In addition, overexpression of c-kit gene in AML is observed in 80–90% of blast cells and exonic point mutations in this gene are identified in 33–45% of AML patients^10,11^. C-KIT mutations frequently affect the external cell surface of the receptor where exon 8, and tyrosine kinase area exon 17 is present. Neoplasms disturbing the juxta-membrane territory where exon 10 and 11 is present are less common. c-KIT modifications represent poor outcome in patients of CBF-AML^7^.

Heterogeneous mutations in exon 8 involve small insertions/deletions affecting amino acid 417–419. A well depicted mutant of exon 8 is c-Kit^T417IΔ418–419^ where isoleucine replaces threonine at position 417 with a small deletion of 418–419^12^. Mutations in exon 11 of c-kit gene are observed in CBF-AML, human cell tumor, gastrointestinal tumor and adenoid cystic tissue. In AML, excessive expression c-kit gene has been reported in 60–80% patients and missense mutations carry 33–45% weightage of AML cases^13,14^. Very rarely cytogenetically normal AML patients have been reported to express alterations in c-kit exon 11^15^. Exon 17 alterations are also reported in similar studies in abnormal karyotype AML. Most observed alterations in this exon fall between amino acid residues 816–822 and are linked to unfavorable prognosis^16^.

This study focuses on screening of c-kit exon 8, 11 and 17 mutations in newly diagnosed AML patients including cytogenetically normal cases, to investigate likelihood of occurrence of these changes irrespective of the cytogenetic status.

## Materials and methods

### Patients and ethical statement

Blood samples of newly diagnosed AML patients (n = 31) age ranging between 16 and 65 years, were collected from different tertiary care hospitals of Lahore, Pakistan including Jinnah Hospital, Lahore and Mayo Hospital, Lahore; a written informed consent was obtained from all the study participants. Blood from healthy individuals (n = 10) was also collected as control group. The study was approved by the ethics review committee, Division of Science and technology, University of education township, Lahore. AML was defined on basis of French-American-British (FAB) classification. It have been confirmed that the experimental samples, including the collection, complied with relevant institutional, national, and international guidelines and legislation with appropriate permissions from authorities of the Jinnah Hospital, Lahore and Mayo Hospital, Lahore, Pakistan. The blood samples were collected in EDTA tubes and transported to research laboratory within 02 h, where it was stored below 20 °C. Patient data including clinical and hematological parameters was noted on structured data forms. Patient characteristics at the time of diagnosis, are given in Table 1. Twenty months follow up of the selected cohort was performed to document their response towards the treatment.

**Table 1:** Clinical features of AML patients (n = 31) included in the present study.

Clinical measures	Features	No. of patients n = 31 (%)
Gender	Males	21 (67.7%)
	Females	10 (32.2%)
Age	< 30	15 (48.4%)
	> 30	16 (51.6%)
Symptoms at diagnosis	Fever	25 (80.6%)
	Flu	7 (22%)
	Sore throat, cough	4 (12.9%)
	Spot on body	1 (3.2%)
	Swelling	2 (6.5%)
	Breathless	2 (6.5%)
	Bleeding gums	2 (6.5%)
	Pregnancy	2 (6.5%)
	Anemic	5 (16.1%)
FAB Subtype	M1	2 (6.5%)
	M2	5(16.1%)
	M3	3 (9.7%)
	M4	9 (29%)
	M5	7 (22.6%)
	M6	2 (6.5%)
	M7	2 (6.5%)
	Missing data	1 (3.2%)
Cytogenetic status	Abnormal t(8:21)	2 (6.5%)
	Normal	18 (58%)
Liver size	Enlarged	16 (51.6%)
	Normal	14 (45.2%)
	Missing data	1 (3.2%)
Spleen size	Enlarged	12 (38.7%)
	Normal	18 (58%)
	Missing data	1 (3.2%)
Platelet count (10e9/L)	< 150	25 (80.6%)
	> 150	5 (16.1%)
	Missing data	1 (3.2%)
Total leukocyte count (10e9/L)	< 10	10 (61.3%)
	10–40	11 (35.5%)
	Missing data	1 (3.2%)
Hemoglobin (g/dL)	< 10	24 (77.4%)
	> 10	6 (19.4%)
	Missing data	1 (3.2%)
Blast cells (%)	< 60	28 (90.3%)
	> 60	2 (6.5%)
	Missing	1 (3.2%)

### DNA extraction, exon amplification and Sequencing

DNA was extracted from wholeblood using QIAamp DNA mini kit (Qiagen Cat # 51304) according to manufacturer’s instruction. Qualitative and quantitative analysis was performed and DNA was stored at – 20 °C until further process. Following primer sets were used to carry out amplification of exon 8 (Forward 5′-ACCACAGTCCATGCCATCA-3′ Reverse 5′-ACCACAGTCCATGCCATCA-3′) exon 11 (Forward 5′-ACCACAGTCCATGCCATCA-3′ Reverse 5′-TCCACCACCCTGTTGCTGTA-3′) and exon 17 (Forward 5′-TGGTGTACTGAATACTTTAAAACAAAA-3′ Reverse 5′- TGCAGGACTGTCAAGCAGAG-3′) of c-Kit gene. Extracted DNA (20 ng/µl) for each sample, along with three sets of primers were sent to Zixi biotechnology company Co., Ltd. China, for amplification of selected DNA sites and their bidirectional Sanger Sequencing.

### Sequence analysis

The sequenced templates were analyzed using Geneious Prime 2019-Sequence analysis software. The sequenced exons 8, 11 and 17 of healthy individuals from Pakistani population were compared to NCBI GenBank accession number: X06182.1 to identify any discrepant sites as population variants. Such variants are noted to be excluded while analyzing patient sequences and are not considered mutations. Patient sequences were arranged individually to the reference sequence and were screened to identify any mismatching sites.

### Statistical analysis

The association of clinical and hematological parameters of AML patients with their mutation status was analyzed through Chi Square test of association (Fischer’s exact test) IBM SPSS version 19. A p-value below 0.05 was considered significant, statistically. The strength of association was assessed by Phi and Cremer’s V.

### In silico analysis

SWISS-MODEL was used for the homology modeling of wild and mutant types of c-kit protein. It generates the 3D structures of proteins and also provides some parameters like GMQE, QMEAN Z-score for quality assessment of the generated model.

### Validation

ProtParam was used for the computation of the physical and chemical parameters of the given proteins. It is proficient to compute these parameters for the protein models generated by SWISSMODEL or for the provided protein sequences by user. Another tool VADAR is used for the quantitative and qualitative assessment of the proteins of c-kit gene. While ProSA web is an online tool which was used for the identification of an error in 3D model of protein which was obtained from Swiss model. For further validation Molprobity was used and Ramachandran plot was obtained. The input for this online tool should be in PDB format which was obtained from Swiss model for both wild type and mutant.

### 3D structural analysis

UCSF Chimera was used for visualization and analysis of protein structures. The structure of wild type and mutant was super imposed to identify the amino acid alteration.

### Functional analysis

Sorting intolerant from tolerant (SIFT) was used to calculate the affect of amino acid replacement in protein function or not. The SIFT score < 0.05 was expected to be damaging or deleterious, otherwise it is considered tolerant https://sift.bii.a-star.edu.sg/sift-bin/SIFT_seq_submit2.pl. While PolyPhen-2 (Polymorphism phenotyping v2) predicts the impact of alteration in residue of protein on function and structure. The results are interpreted as benign or possibly damaging if the values lie between 0 and 9.5 and probably damaging when value is 1) http://genetics.bwh.harvard.edu/ggi/cgi-bin/ggi2.cgi. PROVEAN, an online in silico functional analysis tool was used for the calculation of impact of amino acid substitution on function of protein. If the score is > 2.5 the affect is neutral, while the score is ≤ 2.5 the affect is deleterious http://provean.jcvi.org/provean_seq_report.php?jobid=2063533623555610.

## Results

**Figure 1:**
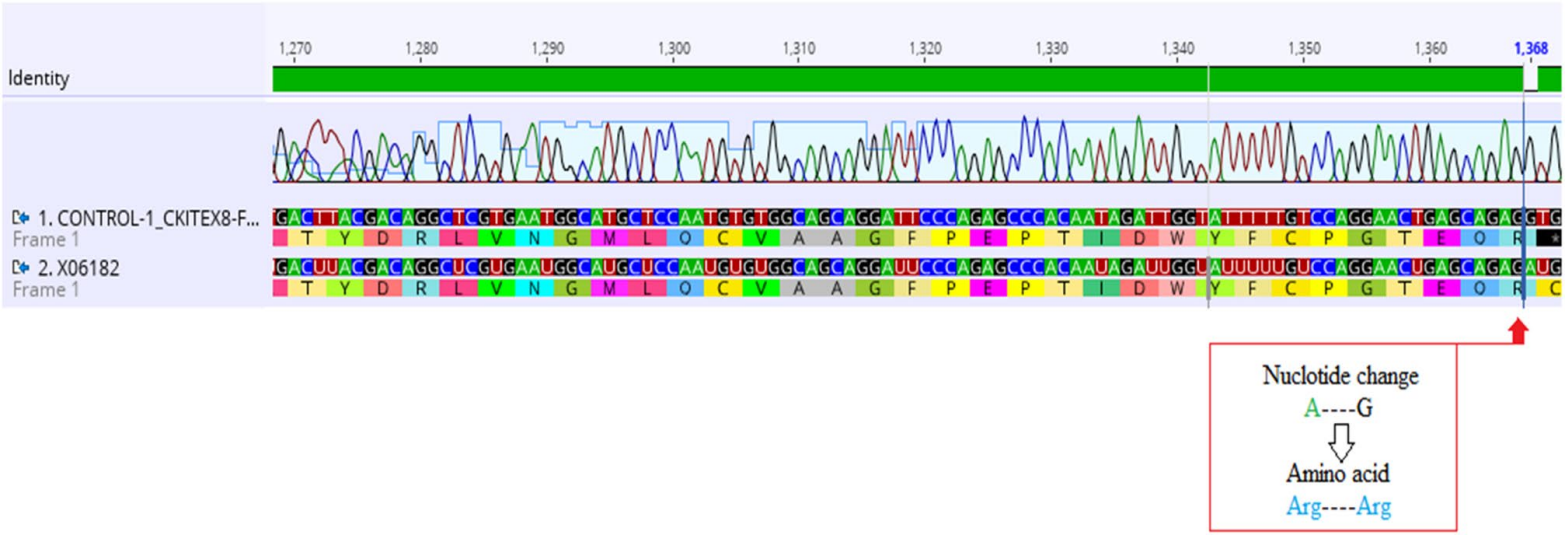
Electropherogram showing nucleotide change (A → G) in Control (healthy person) at position 1368 in c-kit exon 8. However resulting amino acid after this change remains the same as in the reference sequence (GenBank: X06182), which indicates discrepant site in c-kit exon 8.

All three exons of c-kit gene were successfully amplified and sequenced bidirectional. The screening of sequences of healthy individuals showed a mismatching nucleotide at position 1368 (exon 8) when aligned against the reference sequence (NCBI GenBank accession number: X06182.1). However, the resulting amino acid remains same i.e. Arg499Arg. This change is consistent in all patient sequences and was excluded in reporting mutations among AML patients (Fig. 1). A total of fifteen out of 31 patients (48.4%) were observed to have one, two or more mutated sites. One patient exhibited mutations in all 3 studied exons, one had mutations in exon 8 and 11 while one patient manifested nucleotide alterations in exon 8 and 17. Rest of 12 mutated AML patients harbored mutations only in exon 8 of c-kit gene.

### C-kit gene exon 8 mutations

**Figure 2:**
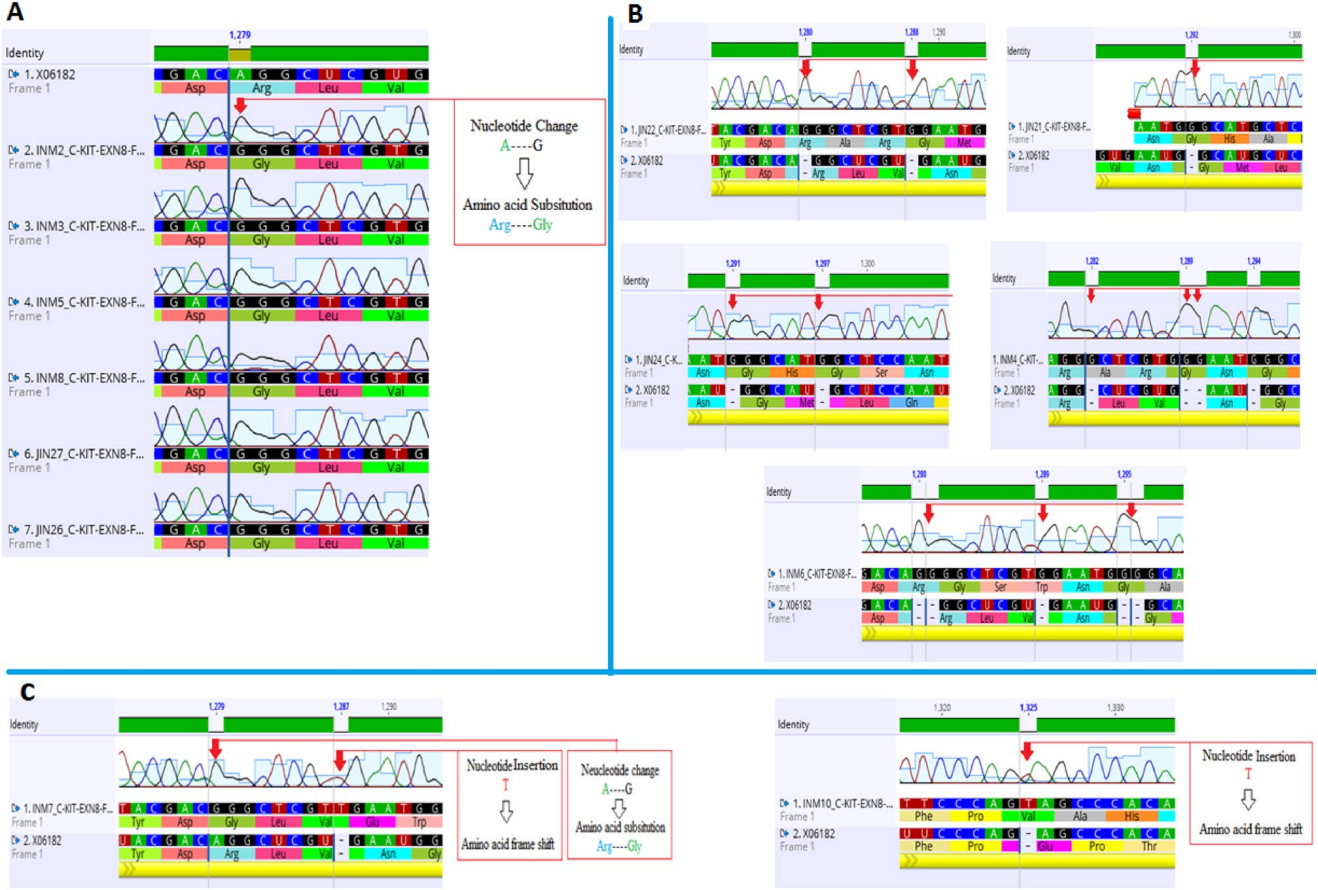
Electropherograms showing alignment of c-kit exon 8 in AML patient sequences against NCBI GenBank accession number: X06182.1. Multiple alignment of patient sequences (n = 9) harboring nucleotide substitution 1279 A > G resulting in Arg420Gly (**A**). Insertion of nucleotide Guanine (G) at different positions in different individuals (n = 05) as a result frame of translating amino acid is shifted (**B**). Substitution 1279 A > G in one and insertion of Thymine (T) at positions 1287 and 1325 in another patient resulting into frame shift (**C**).

All mutated patients (48.4%) had nucleotide changes in exon 8. Out of which 9 (60%) were observed to harbor amino acid substitution Arg420 by Gly due to nucleotide change of Adenine by Guanine at position 1279 (Fig. 2A). Five Patients (33.33%) were observed to have insertion of nucleotide G at positions 1280, 1282, 1288, 1289, 1291, 1292, 1295, 1294, 1297and 1393 either once, twice or thrice, as a result of these insertions frame was shifted and resulting amino acid sequence varied from wild type (Fig. 2B; Table 2). Two patients (13.33%) were observed with insertion of nucleotide T at position 1287 and 1325 causing frame shift (Fig. 2C). All the detected nucleotide aberrations are novel and are not reported previously in cases of AML.

**Table 2:** C-kit exon 8,11 and 17 mutations detected in the present cohort of Acute myeloid leukemia (n = 31).

Sr #	Patient ID	Gender M/F	Age (Yrs)	Cyt. Abnl	FAB type	Mutation detected in cKit Exon	Nucleotide Change	Aminoacid change	Reported by
1	INM2	F	30	n/a	M2	08	1279A > G	Arg420Gly	Novel
						11	1684T > A	Val555Glu	^17^
2	INM3	M	40	n/a	M5	08	1279 A > G	Arg420Gly	Novel
3	INM4	M	27	n/a	M6	08	Insertion of G at 1282, 1289 and 1294	Amino acid frameshift	Novel
4	INM5	F	24	T(8;21)	M2	08	1279A > G	Arg420Gly	Novel
5	INM6	M	34	n/a	M4	08	Insertion of G at 1280, 1289 and 1295	Amino acid frameshift	Novel
6	INM7	M	21	n/a	M1	08	1279A > G	Arg420Gly	Novel
							Insertion of T at 1287	Amino acid frameshift	Novel
7	INM8	F	29	n/a	M4	08	1279A > G	Arg420Gly	Novel
8	INM10	F	17	T(8;21)	M2	08	Insertion of T at 1325	Amino acid frameshift	Novel
						11	1684T > A	Val555Glu	^17^
						17	2415C > T	Ile798Ile	^18^
9	JIN21	M	38	n/a	M6	08	Insertion of G at 1292	Amino acid frameshift	Novel
						17	2415C > T	Ile798Ile	^18^
10	JIN22	M	52	n/a	M5	08	Insertion of G at 1280 and 1288	Amino acid frameshift	Novel
11	JIN24	M	52	n/a	M1	08	Insertion of G at 1291 and 1297	Amino acid frameshift	Novel
12	JIN25	M	18	n/a	M5	08	1279A > G	Arg420Gly	Novel
13	JIN26	M	18	n/a	M5	08	1279A > G	Arg420Gly	
14	JIN27	M	47	n/a	M4	08	1279A > G	Arg420Gly	
15	JIN28	M	25	n/a	M2	08	1279A > G	Arg420Gly	

*M* Male, *F* Female, *Yrs* Years, *n/a* not any, *Cyt.Abnl* Cytogenetic abnormality, *FAB* French American British classification.

### C-kit gene exon 11and 17 mutations

**Figure 3:**
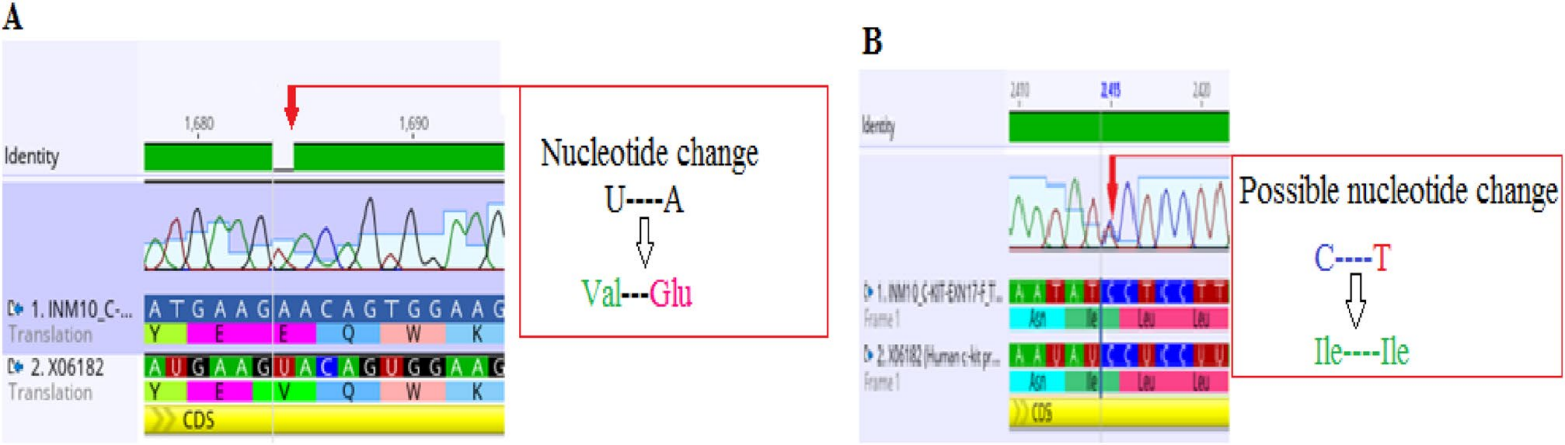
Electropherograms showing 1684 T > A substitution in exon 11 (**A**) and a heterozygous silent mutation 2415 C > T in exon 17 (**B**) when aligned to the reference sequence X06182.1. Both mutations were observed twice in different patients.

Two patients (6.45%) were observed to manifest nucleotide change Thymine to adenine at position 1684, resulting in translation of Glutamic acid555 instead of Valine in exon 11 (Fig. 3A). In addition, analysis of exon 17 revealed heterozygous silent mutation in two patients (6.45%) at position 2415, where the overlapping peak of Thymine over cytosine can be observed, which does not affect the resulting amino acid (Fig. 3B).

### Relationship of mutation status with clinical and hematological parameters

The Chi square test of association suggested that FAB subtypes (x^2^ = 12.524, *p* = 0.029), Spleen size (x^2^ = 4.288, *p* = 0.038) and Red blood cell count (x^2^ = 8.447, *p* = 0.007) vary significantly between Mutant and non-mutant group of patients. Patients with FAB subtypes M3 and M7 manifested no mutations at all, whereas majority of individuals with M2 and M5 were mutated for c-kit gene. Spleen size was observed to be normal in majority of mutants (80%), whereas RBCs were significantly lower than normal (< 4.2 × 106/µl) in all mutant patients. Rest of the clinical and hematological indicators i.e., age, gender, cytogenetic status, liver size, hemoglobin level, Total leukocyte count, Platelet count and Blast cell % did not vary significantly among mutant and wild type AML patients.

### Effect of c-kit mutations on the prognosis of AML

**Figure 4:**
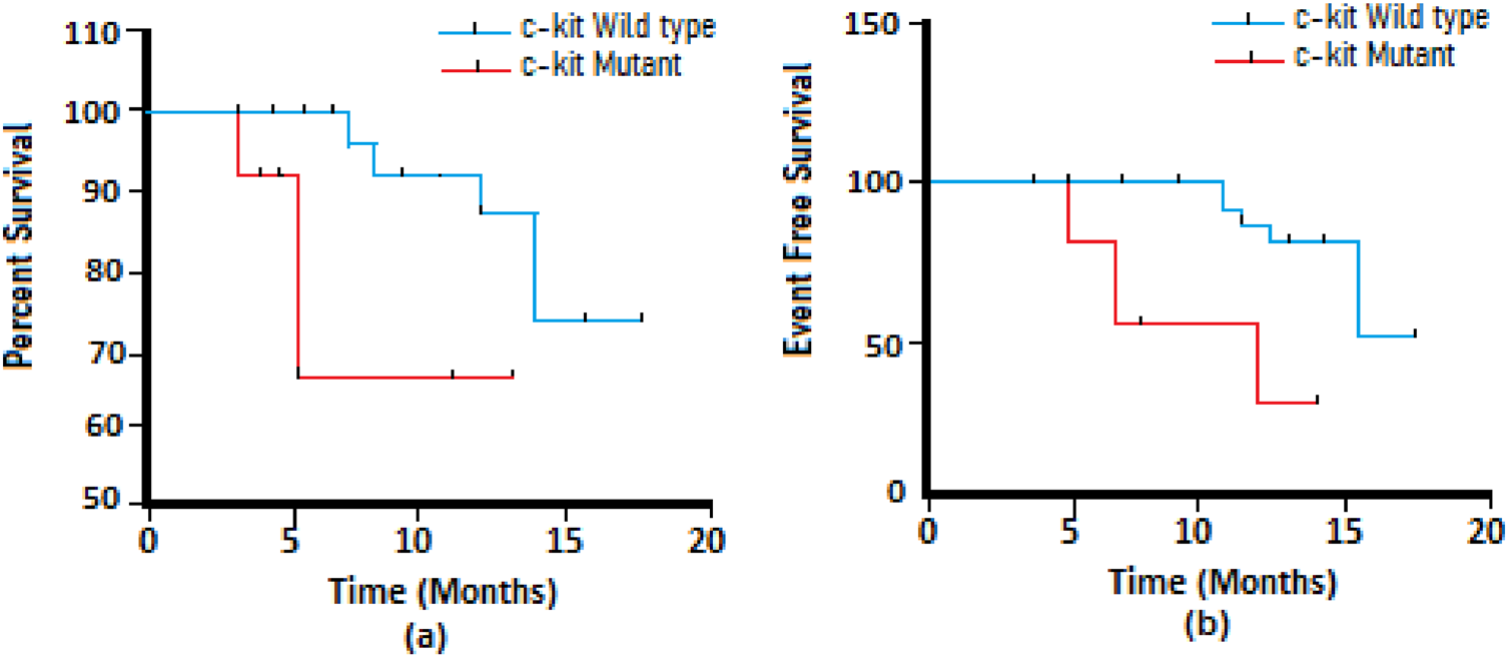
Effect of c-kit mutations on prognosis of AML patients. Low overall (**a**) and Event free survival (**b**) in c-kit mutant and non mutant patients was observed in Kaplan Meier analysis.

C-kits mutation are shown to be a negative predictor of EFS (P = 0.0048) and OS (P = 0.032) employing the Kaplan–Meier analysis. The EFS and OS rates were 30.4% and 48.3% respectively for c-kit mutant patients while it was 38.4% (EFS) and 64.3% (OS) for non-mutant patients (Fig. 4).

### In silico analysis

In current study the in silico analysis was done for Arg420Gly mutation of c-kit gene. The protein homology model was generated by using SWISS MODEL and obtained some information for the quality assessment of the generated model. The quality model energy analysis (QMEAN-Z) score and global model quality estimate (GMQE) values reflected that there were higher agreement between templet and target structure of same size and the alignment was highly accurate. The chemical and physical parameters of the generated model was analyzed by ProtParam and found no significant variation between mutant and wildtype. The VADAR Analysis was used for qualitative and quantitative assessment of protein. Only a slight difference was observed for coils and beta chains of wild and mutant models. Statistics were same for both helices and turns. The values for mean hydrogen bond energy, mean hydrogen bond distance, and the number of residues with hydrogen bonds were also same for both wild and mutant typec-KIT gene proteins.

**Figure 5:**
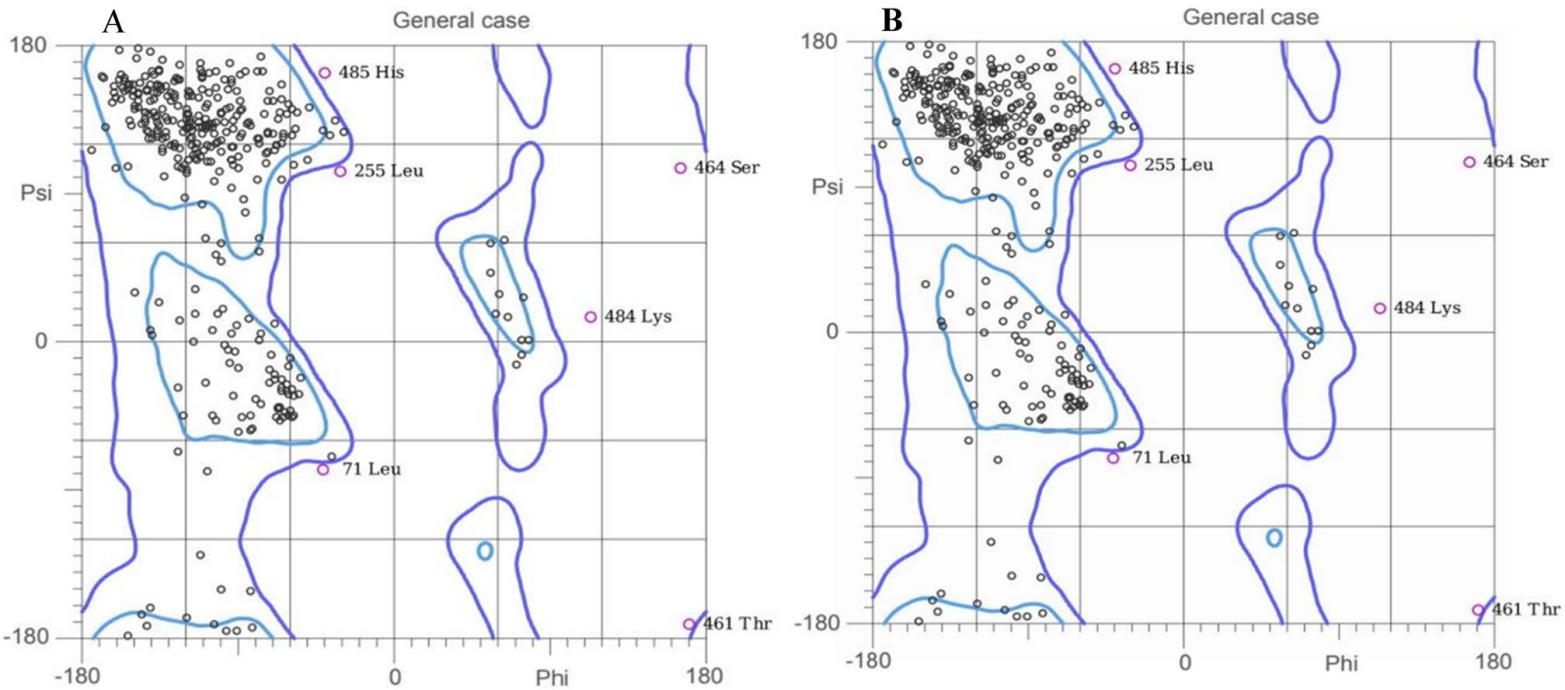
Representing the Ramachandran plot for wildtype c-kit protein model (**A**) and Ramachandran plot for mutant c-kit protein model (**B**) generated by Molprobity.

**Figure 6:**
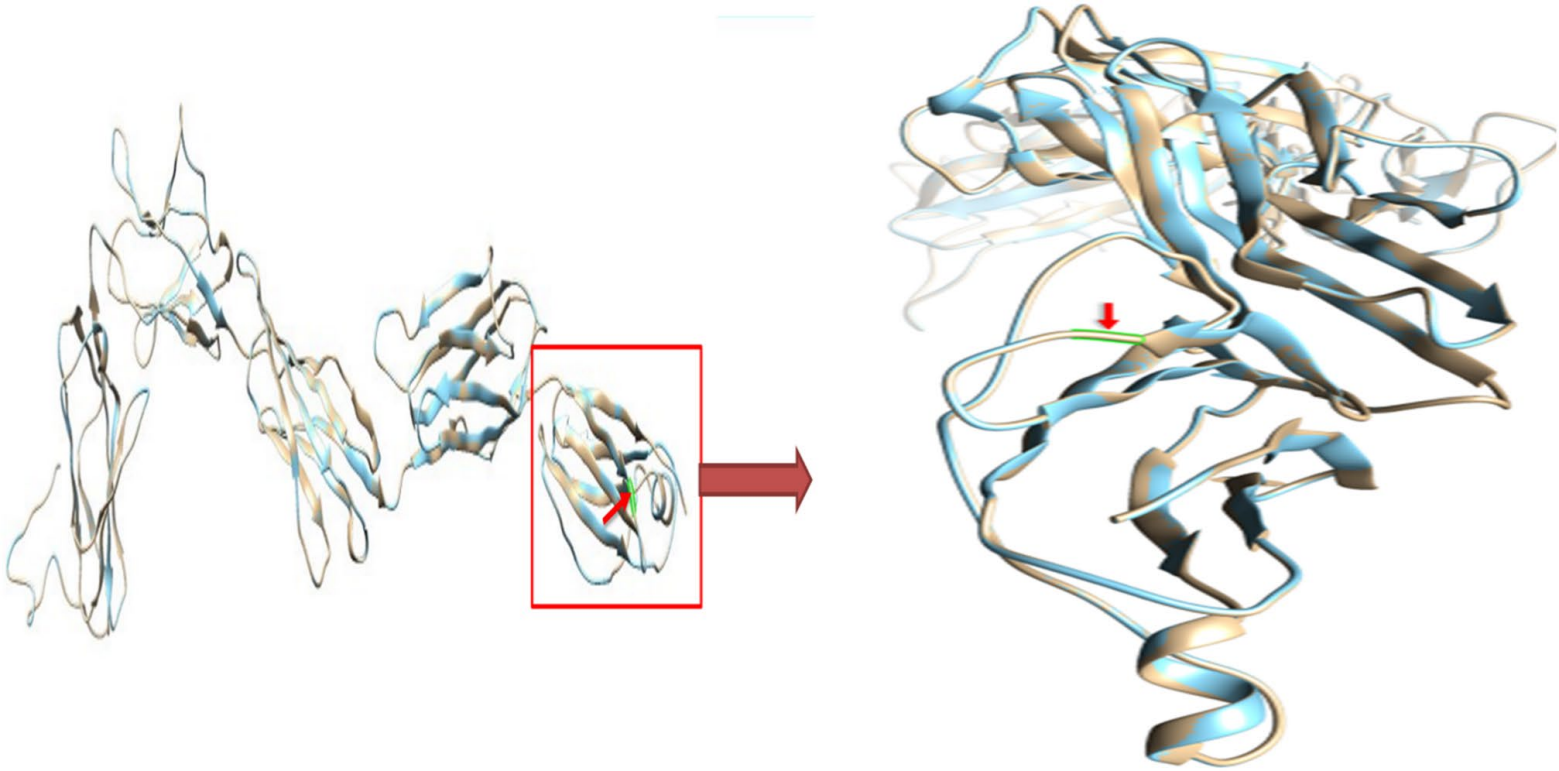
The highlighted region in this figure obtained through UCSF Chimera represents the altered site Arg420Gly in c-kit protein, identified in Acute myeloid Leukemia patients (n = 09).

ProSA web was used for identification of any error in 3D structure of proteins generated by Swiss model. It disclosed that the 3D structure obtained for mutant type was built using 498 amino acid residues. The Z- score for the structure of mutant type was − 7.73 which lies in the characteristic range of native protein showing minimal errors in the structure. While further validation of protein structure was done by MOLPROBITY. The Ramachandran plot were obtained for both mutant and wildtype (Fig. 5A,B). The 88.9% residues were shown to be lying on favored region for both mutant and wildtype. Similarly, 97% residues were falling in allowed region for both mutant and wildtype (Table 3). The superimpose structure of mutant and wildtype was obtained by UCSF Chimera. The highlighted region shown the mutation Arg420Gly (Fig. 6).

**Table 3:** In silico analysis of mutant and wildtype c-kit gene of Acute Myeloid Leukemia patients (n = 31).

Tools	Parameters	Wildtype	Mutant	Expected
	Total number of amino acids	976	976	
	Number of amino acids in model	498	498	
Swiss model	Template Query number	2ec8.1.A	2ec8.1.A	
	Sequence identity	100	99.81	
	GMQE	0.37	0.37	
	QMEAN	− 4.15	− 4.22	
	Number of amino acids	976	976	
ProtParam	Molecular weight	109,864.57	109,765.43	
	Theoretical pI	6.45	6.45	
	Helix	6	6 (1%)	
	Beta	301	300 (60%)	
	Coils	191	192 (38%)	
Vadar analysis	Turns	52	52 (10%)	
	Mean h-bond distance	2.2 ± 0.3	2.2 ± 0.3	2.0 ± 0.4
	Mean h-bond energy	− 2.0 ± 0.9	− 2.0 ± 0.9	− 2.0 ± 0.8
	Number of residues with H bond	285 (57%)	285 (57%)	373 (75%)
	Z score	− 7.72	− 7.73	
ProSA web	Chain	A	A	
	Amino acids	498	498	
	Percentage of all residues in favored regions	88.9% (441/496)	88.9% (441/496)	
Molprobity	Percentage of all residues in allowed region	97.0% (481/496)	97.0% (481/496)

The functional analysis was done by SIFT, PolyPhen-2, and PROVEAN which predicted the effect of mutation as tolerated, possibly damaging and neutral effect respectively (Table 4).

**Table 4:** Functional analysis of mutant c-kit protein (Arg420Gly) identified in acute myeloid leukemia patients (n = 09).

Substitution	Tools	Prediction	Score
	SIFT	Tolerated	0.48
Arg420Gly	PolyPhen-2	Possibly damaging	0.931
	PROVEAN	Neutral	− 0.607

## Discussion

This is the very first attempt from Pakistan to screen AML patients for c-kit gene mutations bringing eight (08) novel nucleotide alterations to light. This includes single nucleotide replacement as well as insertions of one or more nucleotide bases within the gene sequence. Consistent with the other studies^14,17,18^, we detected majority of mutations (48.38%) in c-kit exon 8 in present investigation, that effect the extracellular portion of kit-receptor^19^. However, exon 11 and 17 mutations were less frequently observed (6.45%), as previous findings also report so^20^. Nine patients were documented to have point mutation Arg420Gly at c-kit exon 8 and one is observed with insertion of guanine (G) at Leu421 which cause frame shift of proceeding amino acid sequence. These two mutations have not been studied previously. However, deletion of this region and nucleotide substitution is reported in gastrointestinal stromal tumor (GIST) and AML patients^21,22^. The amino acid substitution Val555Glu detected in exon 11 of c-kit gene was observed in two patients in present cohort, which was reported as a causative agent of GIST, previously^23^. Studies have reported that most of the mutations in c-kit exon 11 are clumped in codon 550–570^24^. Our results show occurrence of silent heterozygous mutation Ile798Ile in c-kit exon 17 among two AML patients. Earlier^25,26^, reported this heterozygous silent change in Polycythemia vera (PV) and Idopathic myelofibross (IMF) patients, respectively.

In our cohort two patients were cytogenetically abnormal with t(8:21). Both of them were females, one was adult and the other (INM 10) was 17 years of age and harbored mutations in all three sequenced exons i.e., 08, 11 and 12. This interesting finding is supported by the understanding that c-kit gene mutations are more likely to be found in CBF-AML and are attributed to poor prognosis^4,27^. Nevertheless, we have observed a good number of nucleotide changes in cytogenetically normal AML patients 13(42%), which is a rarely reported event^28^. The Overall and event free survival varied among mutant and non-mutant patients regardless of their cytogenetic status in our study, emphasizing the role of genetic changes in poor survival outcomes. This indicates importance of screening for these mutations in cytogenetically normal as well as abnormal AML patients. Deletions and insertions in exon 8 of KIT gene have earlier been described in AML M2 t(8;21) or AML M4Eo inv(16)^21,29–31^. In present consideration, most individuals had FAB subtype M4, whereas mutations were significantly abundant in M2 (26.7%) and M5 (26.7%) subtypes. Prominently, majority of mutant patients were observed to have normal spleen size but lower than normal RBC count. Concordantly, studies describing KIT mutations have associated splenomegaly with occurrence of mutations^5,32^.

In silico analysis of protein structure and parameters of c-kit gene of AML patients suggests no significant variations in physical and chemical parameters of mutant and wildtype. However, the structural analysis reflected the slight difference in structures of mutant and wildtype. The functional analysis was done by SIFT, PolyPhen-2, and PROVEAN which indicated the tolerated, damaging and neutral effects respectively as the result of the replacement of amino acid Arginine by Glycine at position 420. Summarizing the findings of our work, we emphasize that point mutations, deletions and insertions in c-kit proto-oncogene are not necessarily present in cytogenetically abnormal AML but are also observed in patients with normal karyotype. As the detection rate of these mutations is reportedly increased in t(8;21) AML and has gained importance in predicting prognosis, the possibility of their presence in CN-AML and their prognostic impact cannot be neglected.

## Data Availability

All of the datasets generated and/or analysed during the current study are available in the manuscript. The reference sequence was (NCBI GenBank accession number: X06182.1).
